# Frequency of nasal carriers of methicillin-resistant *Staphylococcus aureus* among health personnel in Ayacucho, Peru

**DOI:** 10.17843/rpmesp.2026.432.15609

**Published:** 2026-06-16

**Authors:** Alexandra A. Urrutia, Nilda A. Apayco, Sayda L. Cancho, Liz V. Muñoz, Máximo R. Sosa

**Affiliations:** 1 Universidad Nacional de San Cristóbal de Huamanga, Huamanga, Peru

**Keywords:** Carrier State, Methicillin-Resistant *Staphylococcus aureus*, Health Personnel, Microbial Sensitivity Tests, Cross-Sectional Studies, Peru

## Abstract

**Objective.:**

To estimate the frequency of nasal carriers of Methicillin-Resistant *Staphylococcus aureus* (MRSA) among healthcare personnel of the Red de Salud Huamanga (Ayacucho, Peru) between October and November 2024.

**Materials and methods.:**

Descriptive cross-sectional study in 265 healthcare workers, recruited by convenience sampling. Nasal swabs were cultured on mannitol salt agar, MRSA was confirmed by cefoxitin disk diffusion (EUCAST v14.0 2024: diameter <22 mm). Antimicrobial susceptibility was evaluated by Kirby-Bauer. The use of personal protective equipment (PPE) was recorded by structured questionnaire. Absolute and relative frequencies were calculated.

**Results.:**

*Staphylococcus aureus* was detected in 14.0% (37/265) of the participants. Of these isolates, 64.9% (24/37) were MRSA, representing a frequency of 9.1% (24/265). Of the total *Staphylococcus aureus* carriers, MRSA isolates were more frequent in interns/trainees (18.9%; 7/37) and nurses (16.2%; 6/37). Outpatient clinics (27.0%; 10/37) and emergency (13.5%; 5/37) concentrated the highest number of carriers. High resistance to erythromycin was observed in MRSA (70.8%) and in methicillin-susceptible *Staphylococcus aureus* (84.6%).

**Conclusions.:**

In the studied sample, approximately one in ten healthcare workers was a nasal carrier of MRSA. Given the convenience sampling, the findings should not be extrapolated as population prevalence. Strengthening the use of PPE, hand hygiene, and microbiological surveillance are priority measures to reduce transmission.

## INTRODUCTION

The nasal cavity of healthy individuals, including health personnel, harbors a diversity of microorganisms that constitute the nasal microbiota ^1^. Although most of these microorganisms are commensals, some can act as opportunistic pathogens under certain conditions. Among these microorganisms, species of the genus *Staphylococcus* stand out, particularly *S. aureus*, due to their pathogenic capacity and clinical relevance ^2^^,^^3^.

*S. aureus* is one of the main causative agents of infections in both the nosocomial and community settings, and is especially problematic in intensive care units, neonatal services, and in patients with risk factors such as burns, trauma, immunosuppression, or neutropenia ^4^^,^^5^. Infections caused by *S. aureus* can originate from cross-contamination through healthcare personnel or from endogenous infections derived from the patient’s own microbiota ^6^.

The problem has been significantly exacerbated by the emergence of methicillin-resistant *S. aureus* (MRSA) strains, complicating antimicrobial treatment and increasing morbidity and mortality. In 2017, the World Health Organization classified MRSA as one of the 12 critical priority pathogens due to its global impact on public health ^1^. Epidemiological data show that MRSA infections present a 64% higher mortality rate compared to those caused by methicillin-susceptible *S. aureus* (MSSA) ^7^.

Healthcare personnel play a fundamental role in the epidemiology of *S. aureus*, by acting as a reservoir and transmission vector, particularly of multidrug-resistant strains ^8^. Asymptomatic nasal colonization among healthcare workers constitutes a significant risk factor for nosocomial dissemination, especially during epidemic outbreaks ^9^. Previous studies in Latin America have documented variable rates of MRSA nasal carriers among healthcare personnel, ranging from 5.0% to 53.9% depending on the region and institutional characteristics ^10^^-^^15^.

In the Peruvian context, data on nasal carriers of MRSA in healthcare personnel are mainly limited to two studies conducted by Garcia *et al.* in a third-level hospital in Lima, which reported a frequency of 13.3% among personnel from the medicine and surgery services ^12^, and the study by Cayllahua Arenas conducted at Hospital Goyeneche in Arequipa ^16^. No previous studies were identified in Andean areas such as Ayacucho, where socioeconomic conditions and resource availability could influence biosecurity practices and infection control, which justifies generating local evidence.

The objective of the present study was to estimate the frequency of nasal carriers of MRSA among healthcare personnel from the Red de Salud Huamanga, Ayacucho, and describe the antimicrobial resistance profile of the recovered isolates.

**KEY MESSAGES**

Motivation for the study. Methicillin-resistant *Staphylococcus aureus* (MRSA) is a critical priority pathogen according to the World Health Organization, and its nosocomial transmission involves healthcare personnel as a reserve oir. Local information in Ayacucho, Peru is scarce, hindering evidence-based control.

Main findings. In 265 workers, the frequency of nasal carriers of MRSA was 9.1% (24/265). Interns/trainees (18.9%) and outpatient clinics (27.0%) concentrated the highest nasal carriers. Resistance to erythromycin reached 70.8% in MRSA and 84.6% in methicillin-susceptible *Staphylococcus aureus*.

Public health implications. This study provides local descriptive data for the Red de Salud Huamanga that can serve as a starting point for institutional surveillance.

## MATERIALS AND METHODS

### Study design and population

A descriptive study was conducted between October and November 2024 among health personnel of the Red de Salud Huamanga, Ayacucho, Peru. The study included personnel from public health facilities of two levels of care: first-level health centers (categories I-3 and I-4), which provide outpatient care for low-to-moderate complexity conditions, and a second-level hospital (category II-E), which provides outpatient and inpatient care.

The sample size was estimated using the proportion formula for a finite population, considering the following parameters: total population N = 800 Network workers; 95% confidence level (Z = 1.96); expected proportion p = 0.133, based on the frequency of nasal MRSA carriers reported in health personnel by Garcia et al. in a tertiary hospital in Lima ^12^; q = 1 − p = 0.867; and a margin of error e = 0.05. The calculation was performed in a Microsoft Excel® v.2021 spreadsheet, yielding a minimum required size of 146 participants.

**Figure 1:**
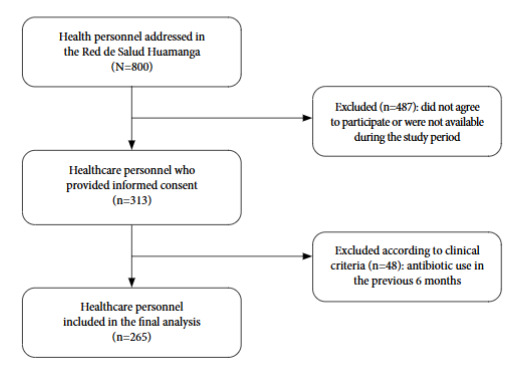
Flowchart of participant selection.

Out of a total of 800 potentially eligible workers, 313 provided written informed consent; after applying the exclusion criteria (did not agree to participate or were not available during the study period, antibiotic use in the previous 6 months, medical conditions affecting the nasal mucosa, or pregnancy), 265 were included in the final analysis (figure 1), exceeding the minimum required size.

### Variables

The variables included in this study are detailed below, grouped by category.

*Main variable*

The MRSA nasal carrier status was operationally defined as the recovery of one or more colonies with morphology compatible with *S. aureus* on mannitol salt agar (yellow colonies with medium acidification), confirmed by positive catalase and coagulase tests, and with an inhibition zone diameter against cefoxitin (30 µg) <22 mm according to EUCAST v14.0 2024 breakpoints. The classification was qualitative (carrier/non-carrier); the recovery of at least one confirmed colony was sufficient to classify a participant as a carrier.

*Sociodemographic variables*

Age (years old; recategorized into ≤33 and ≥34 years), sex (male/female). Age categorization was based on the data (division by the median).

*Occupational variables*

Information was collected on the participants’ role/profession, classifying them as physicians, registered nurses, nursing technicians, obstetricians, biologists, dentists, psychologists, pharmacists, social workers, administrative staff, cleaning staff, and interns/practitioners. The latter corresponded to final-year students of health sciences programs, such as medicine, nursing, obstetrics, dentistry, and biology, who performed care activities under supervision as part of their clinical internship. Likewise, the work area was recorded, categorized as emergency, operating room, outpatient clinic, laboratory, pharmacy, hospitalization, and other services, which included growth and development control (CRED), triage, and immunization. Finally, the length of work experience was grouped into three categories: less than one year, between one and five years, and more than five years.

*Behavioral and exposure variables*

These variables were included because the literature consistently identifies them as factors associated with nasal carriers of *S. aureus* in healthcare personnel (PPE use, hand hygiene, contact with invasive medical devices, contact with high-risk areas) ^12^^,^^18^. These variables were collected using a structured self-report questionnaire previously tested on five healthcare workers not included in the analysis to verify its comprehensibility: Frequency of personal protective equipment (PPE) use (4-level ordinal: “Always,” “Frequently,” “Sometimes,” “Rarely”); frequency of hand hygiene (3-level ordinal: “Always,” “Frequently,” “Sometimes”); use of mask, gloves, and gown (yes/no); use of alcohol-based hand sanitizer (yes/no); reported contact with high-risk areas of microbiological exposure (yes/no); and reported contact with invasive medical devices (yes/no).

### Microbiological Procedures

*Collection of samples, culture and identification*

Nasal swabs were obtained from both nostrils using a single sterile cotton-tipped swab with a wooden shaft. The swab was gently inserted and rotated in each nostril to ensure adequate sampling ^17^. Samples were placed in sterile 15 mL Falcon tubes containing 1 mL of 0.9% sterile saline solution and transported to the laboratory (Laboratorio de Inmunologia y Microbiologia Clinica [Y-207] de la Universidad Nacional de San Cristobal de Huamanga) in a thermal container maintained at approximately 4 °C. The maximum time between sample collection and plating did not exceed 5 hours. No pre-enrichment broth was used; samples were directly plated onto mannitol salt agar (MSA) using the streak plate method. Plates were incubated at 37 °C and evaluated at 18 h; those with scarce or equivocal growth were reincubated and re-evaluated at 24 h. Cultures without characteristic growth after 72 h were considered negative. Isolates were considered *S. aureus* if they produced yellow colonies with a change in medium color (mannitol fermentation), and confirmed as positive by catalase and coagulase tests ^19^. A minimum of one colony (≥1 CFU) with typical morphology confirmed by biochemical tests was sufficient to classify a participant as a carrier.

*Antimicrobial susceptibility tests*

*S. aureus* isolates were evaluated on Mueller-Hinton agar by the Kirby-Bauer disk diffusion method. Bacterial suspensions were standardized to a 0.5 McFarland turbidity standard, and plates were incubated at 37 °C for 18 h. Antimicrobial susceptibility was evaluated using Multodiscs Staph (Liofilchem®, ref. 95260), a multi-antibiotic disk system containing the following agents: cefoxitin, levofloxacin, netilmicin, tetracycline, erythromycin, gentamicin, rifampicin, and linezolid. Inhibition zone diameters were measured and interpreted according to the clinical breakpoints in the European Committee on Antimicrobial Susceptibility Testing (EUCAST) v14.0 (2024) guidelines. Quality control was performed using *S. aureus* ATCC 25923 as the reference strain for disk diffusion, following EUCAST recommendations. Zone diameters for each antibiotic were verified within acceptable ranges before interpreting the results of clinical isolates. MRSA was phenotypically confirmed according to the cefoxitin disk result; isolates with an inhibition zone diameter <22 mm were classified as MRSA, according to EUCAST 2024 criteria. D-test (erythromycin/clindamycin) was not performed for inducible clindamycin resistance detection.

### Statistical analysis

Data processing and analysis was performed using IBM SPSS Statistics for Windows, version 27.0.1 (IBM Corp., Armonk, NY, United States). Quantitative variables were summarized using measures of central tendency and dispersion, while categorical variables were described using absolute (n) and relative (%) frequencies. Considering the cross-sectional design of the study and the participant selection strategy, the results are presented as a description of the included sample, without performing inferential analyses.

### Ethical considerations

The study was approved by the Institutional Ethics Committee of the Universidad Nacional de San Cristóbal de Huamanga, through Certificate N.° 001-2025/CEI-VRI-UNSCH-Ayacucho. The study was conducted in accordance with the ethical principles of the Declaration of Helsinki for research involving human subjects. Written informed consent was obtained from all participants. Data confidentiality was ensured through anonymized coding and password-protected databases.

## RESULTS

Of the 800 health workers contacted, 313 gave informed consent; after applying the exclusion criteria, 265 were included in the final analysis. Nasal carriage of *S. aureus* was observed in 14.0% (37/265). The mean age was 36.1 years (range 19-69), with a predominance of females (77.0%). 41.5% had less than one year of work experience. Interns/trainees showed the highest frequency of nasal carriers of *S. aureus* relative to the total participants (4.2% of the total sample, 11/265), while no nasal carriers were observed among pharmacists, social workers, and cleaning staff (table 1).

**Table 1:** Sociodemographic, occupational, and biosafety characteristics of the studied sample, according to nasal carriage status of *S. aureus*.

Characteristic		Total (n=265)	***S. aureus* + (n=37)**	***S. aureus* - (n=228)**
		n	n (%)	n (%)
Sex				
	Female	204	29 (14.2)	175 (85.8)
	Male	61	8 (13.1)	53 (86.9)
Age (years)				
	≤33	135	21 (15.6)	114 (84.4)
	≥34	130	16 (12.3)	114 (87.7)
Role/Profession				
	Intern/Trainee	72	11 (15.3)	61 (84.7)
	Nurse	46	7 (15.2)	39 (84.8)
	Nursing technician	37	6 (16.2)	31 (83.8)
	Obstetrician	22	5 (22.7)	17 (77.3)
	Physician	21	1 (4.8)	20 (95.2)
	Biologist	19	1 (5.3)	18 (94.7)
	Dentist	13	3 (23.1)	10 (76.9)
	Pharmacist	12	0 (0.0)	12 (100.0)
	Administrative	9	1 (11.1)	8 (88.9)
	Psychologist	7	2 (28.6)	5 (71.4)
	Social worker	5	0 (0.0)	5 (100.0)
	Cleaning staff	2	0 (0.0)	2 (100.0)
Work Area				
	Emergency	51	7 (13.7)	44 (86.3)
	Operating room	1	0 (0.0)	1 (100.0)
	Outpatient clinic	84	13 (15.5)	71 (84.5)
	Laboratory	37	4 (10.8)	33 (89.2)
	Pharmacy	14	0 (0.0)	14 (100.0)
	Hospitalization	7	3 (42.9)	4 (57.1)
	Others^a^	71	10 (14.1)	61 (85.9)
Frequency of PPE use				
	Always	137	17 (12.4)	120 (87.6)
	Frequently	93	10 (10.8)	83 (89.2)
	Sometimes	28	7 (25.0)	21 (75.0)
	Rarely	7	3 (42.9)	4 (57.1)
Frequency of hand hygiene				
	Always	230	28 (12.2)	202 (87.8)
	Frequently	26	8 (30.8)	18 (69.2)
	Sometimes	9	1 (11.1)	8 (88.9)
Other practices (Yes)				
	Use of hand sanitizer	253	34 (13.4)	219 (86.6)
	Use of mask, gloves and gown	133	13 (9.8)	120 (90.2)
	Contact with high-risk areas	17	2 (11.8)	15 (88.2)
	Contact with medical devices	90	9 (10.0)	81 (90.0)

PPE: personal protective equipment.

The percentages are calculated on the total of each row.

a The Other category includes growth and development control (CRED), triage, and immunization.

### Distribution according to role/profession and area of work

Among carriers, 64.9% were MRSA, which represents a nasal carrier frequency of 9.1% of the total sample studied. Table 2 characterizes the 37 *S. aureus* carriers according to MRSA/MSSA status. MRSA nasal carriers among the 37 carriers followed a similar pattern, with interns/trainees (7/37, 18.9%) and nurses (6/37; 16.2%) as the most represented groups (Table 2).

**Table 2:** Distribution of *S. aureus* carriers according to methicillin-resistant *S. aureus* / methicillin-susceptible *S. aureus* status and sociodemographic and occupational characteristics (n=37).

Characteristic		Carriers n	MRSA n (%)	MSSA n (%)
Sex				
	Female	29	20 (69.0)	9 (31.0)
	Male	8	4 (50.0)	4 (50.0)
Age (years)				
	≤33	21	14 (66.7)	7 (33.3)
	≥34	16	10 (62.5)	6 (37.5)
Role/Professional				
	Intern/Trainee	11	7 (63.6)	4 (36.4)
	Nurse	7	6 (85.7)	1 (14.3)
	Nursing Technician	6	2 (33.3)	4 (66.7)
	Obstetrician	5	4 (80.0)	1 (20.0)
	Physician	1	1 (100.0)	0 (0.0)
	Biologist	1	1 (100.0)	0 (0.0)
	Dentist	3	2 (66.7)	1 (33.3)
	Administrative	1	0 (0.0)	1 (100.0)
	Psychologist	2	1 (50.0)	1 (50.0)
Work Area				
	Emergency	7	5 (71.4)	2 (28.6)
	Outpatient clinic	13	10 (76.9)	3 (23.1)
	Laboratory	4	2 (50.0)	2 (50.0)
	Hospitalization	3	3 (100.0)	0 (0.0)
	Others^a^	10	4 (40.0)	6 (60.0)

MRSA : Methicillin-resistant *S. aureus*, MSSA: Methicillin-susceptible *S. aureus*.

The percentages are calculated on the total of each row.

a The Other category includes growth and development control (CRED), triage and immunization.

### Antimicrobial resistance profile

Antimicrobial susceptibility tests revealed variable resistance profiles between MRSA and MSSA isolates (table 3). Both groups exhibited high rates of resistance to erythromycin (70.8% in MRSA; 84.6% in MSSA). Resistance to tetracycline was higher in MSSA (53.8%) compared to MRSA (25.0%). In contrast, MSSA isolates showed higher resistance to linezolid (53.8%) compared to MRSA (29.2%) and gentamicin (29.2% versus 30.8% in MSSA). Resistance to rifampicin remained low in both groups (<10%), while resistance to levofloxacin was similar (29.2% in MRSA; 30.8% in MSSA).

**Table 3:** Comparison of antibiotic resistance based on evaluated antibiotics between methicillin-resistant *S. aureus* and methicillin-susceptible *S. aureus*.

Antibiotic	**Methicillin-resistant *S.aureus* (n=24)**		**Methicillin-susceptible *S. aureus* (n=13)**	
	Resistant n (%	Susceptible n (%)	Resistant n (%)	Susceptible n (%)
Linezolid (LNZ)^a^	7 (29.2)	17 (70.8)	7 (53.8)	6 (46.2)
Rifampicin (RD)	2 (8.3)	22 (91.7)	1 (7.7)	12 (92.3)
Gentamicin (CN)	7 (29.2)	17 (70.8)	4 (30.8)	9 (69.2)
Erythromycin (ERY)	17 (70.8)	7 (29.2)	11 (84.6)	2 (15.4)
Tetracycline (TE)	6 (25.0)	18 (75.0)	7 (53.8)	6 (46.2)
Netilmicin (NET)	3 (12.5)	21 (87.5)	5 (38.5)	8 (61.5)
Levofloxacin (LEV)	7 (29.2)	17 (70.8)	4 (30.8)	9 (69.2)

Susceptibility was evaluated by Kirby-Bauer disk diffusion using Multodiscs Staph (Liofilchem®, ref. 95260), interpreted according to EUCAST 2024 clinical breakpoints (v.14.0).

MRSA was defined by zone diameter <22 mm against cefoxitin.

Percentages are calculated within each subgroup (R + S = 100% for MRSA [n=24] and MSSA [n=13] separately).

a The rates of linezolid resistance were not confirmed by reference method (broth microdilution / E-test).

### Infection control practices and colonization

Among the 37 carriers, the distribution of self-reported infection control practices showed that a higher frequency of carriers reported inconsistent use of PPE. Within the intern/practitioner group, 54.5% (6/11) reported always using PPE (table 4).

**Table 4:** Frequency of use of personal protective equipment among participants positive for *S. aureus*.

Role/profession	Always n (%)	Frequently n (%)	Sometimes n (%)	Rarely n (%)	Total n (%)
Intern/Trainee	6 (54.5)	2 (18.2)	3 (27.3)	0 (0.0)	11 (29.7)
Nursing technician	4 (66.7)	1 (16.7)	0 (0.0)	1 (16.7)	6 (16.2)
Dentist	2 (66.7)	1 (33.3)	0 (0.0)	0 (0.0)	3 (8.1)
Administrative	0 (0.0)	0 (0.0)	0 (0.0)	1 (100.0)	1 (2.7)
Biologist	1 (100.0)	0 (0.0)	0 (0.0)	0 (0.0)	1 (2.7)
Nurse	4 (57.1)	2 (28.6)	1 (14.3)	0 (0.0)	7 (18.9)
Physician	0 (0.0)	0 (0.0)	0 (0.0)	1 (100.0)	1 (2.7)
Obstetrician	0 (0.0)	3 (60.0)	2 (40.0)	0 (0.0)	5 (13.5)
Psychologist	0 (0.0)	1 (50.0)	1 (50.0)	0 (0.0)	2 (5.4)
Total	17 (45.9)	10 (27.0)	7 (18.9)	3 (8.1)	37 (100.0)

The percentages are calculated on the total of each row and the percentages of the Total column are calculated on all carriers of *S. aureus* (n=37).

Regarding hand hygiene, 75.7% (28/37) of participants with positive isolation for *S. aureus* reported always performing it, 21.6% (8/37) frequently, and 2.7% (1/37) sometimes. Among nurses, 85.7% (6/7) indicated always performing hand hygiene, while it was 100.0% in biologists, obstetricians, and psychologists (table 5).

**Table 5:** Frequency of hand hygiene among positive cases for *S. aureus* according to role/profession.

Role/profession	Always n (%)	Frequently n (%)	Sometimes n (%)	Total n (%)
Intern/Trainee	8 (72.7)	3 (27.3)	0 (0.0)	11 (29.7)
Nursing technician	4 (66.7)	2 (33.3)	0 (0.0)	6 (16.2)
Dentist	2 (66.7)	1 (33.3)	0 (0.0)	3 (8.1)
Administrative	0 (0.0)	0 (0.0)	1 (100.0)	1 (2.7)
Biologist	1 (100.0)	0 (0.0)	0 (0.0)	1 (2.7)
Nurse	6 (85.7)	1 (14.3)	0 (0.0)	7 (18.9)
Physician	0 (0.0)	1 (100.0)	0 (0.0)	1 (2.7)
Obstetrician	5 (100.0)	0 (0.0)	0 (0.0)	5 (13.5)
Psychologist	2 (100.0)	0 (0.0)	0 (0.0)	2 (5.4)
Total	28 (75.7)	8 (21.6)	1 (2.7)	37 (100.0)

The percentages are calculated on the total of each row, and the percentages in the Total column are calculated on all *S. aureus* carriers (n=37).

## DISCUSSION

This study describes a frequency of nasal carriers of *S. aureus* of 14.0% among healthcare personnel from the Red de Salud Huamanga, with an MRSA frequency of 9.1% in the studied sample. Likewise, a higher frequency of MRSA nasal carriers was observed among interns and trainees (18.9%), a higher concentration of carriers in outpatient clinics (27.0%), and among personnel who reported inconsistent use of PPE.

The overall frequency of nasal carriers of *S. aureus* (14.0%) was lower than that reported in Paraguay (26.6%) ^20^ and Uruguay (24%) ^21^. The differences may reflect population characteristics, institutional infection control protocols, or methodological variations. However, the frequency of MRSA among carriers (64.9%) was markedly higher than in other Latin American settings, suggesting strong antimicrobial selective pressure in the region. The frequency of nasal carriers of MRSA (9.1%) is comparable with Ecuador (12.5%) ^22^, but lower than Paraguay (23.8%) ^20^. In contrast, studies in Brazilian primary care units reported prevalences exceeding 50% ^10^, possibly reflecting differences in care levels or in the community burden of MRSA.

Interns/trainees were the group with the highest frequency of nasal carriers, followed by nurses. This is consistent with Colombian data, where interns were also the most colonized group (33.8%) ^23^, but contrasts with Argentinian studies where physicians predominated ^11^. Factors may include limited experience in biosafety protocols, high burden of direct patient contact, frequent rotation between services, and weaker supervision. Women represented 77.0% of the participants, consistent with the feminization of nursing and obstetrics in Peru ^24^^-^^26^. Although 78.4% of positive cases were women, this reflects the composition of the workforce and not a sex-associated susceptibility.

Adherence to basic biosafety practices was suboptimal. The Pan American Health Organization highlights hand hygiene as the most effective strategy against MRSA ^27^. In Peru, Cayllahua Arenas ^16^ found a lower frequency of carriers among personnel who washed their hands >10 times per shift. Evidence also supports the importance of technique and the use of chlorhexidine and brush-less friction as determinants of bacterial load reduction ^28^. Low compliance among interns may be related to workload, resource limitations, and insufficient reinforcement, which underscores the need for targeted training within this group.

In the studied sample, the frequency of MRSA descriptively concentrated in outpatient clinics and emergency services more than in hospitalization. Unlike studies that identify intensive care units as critical points ^29^, this distribution could reflect a high outpatient flow, the presence of asymptomatic community carriers ^30^ or a differential application of biosecurity among services. Venezuelan research reported higher carriers in internal medicine and surgery (30.4%) ^9^, while studies in Lima showed lower prevalence in pediatrics and gynecology (2.1%) compared to medicine and surgery (13.3%) ^12^. The variability among services highlights the need for differentiated surveillance.

Resistance profiles revealed worrisome multidrug resistance. Among MRSA isolates, 70.8% were resistant to erythromycin, 29.2% to linezolid, gentamicin, and levofloxacin, 25.0% to tetracycline, 12.5% to netilmicin, and 8.3% to rifampicin. These findings are consistent with Venezuelan (52.9% resistance to erythromycin) ^8^ and Brazilian (49.2% resistance to erythromycin) ^10^ studies. High resistance to erythromycin in both MRSA (70.8%) and MSSA (84.6%) suggests selective pressure from macrolide use. Linezolid resistance rates in this study (29.2% in MRSA and 53.8% in MSSA) are markedly higher than global surveillance data reporting <1% resistance ^31^^,^^32^. This discrepancy probably reflects methodological factors rather than true resistance. CLSI recently documented that previous disk diffusion breakpoints for linezolid in staphylococci produced errors exceeding 15% ^33^. In this study, the use of Multodiscs Staph (Liofilchem® ref. 95260) instead of individually placed disks may have further compromised accuracy due to potential overlap of inhibition zones. These linezolid resistance data should not be used to guide empirical therapy until confirmed by broth microdilution and molecular characterization.

Likewise, a higher frequency of carriers was observed among staff who reported performing hand hygiene “frequently” compared to those who indicated doing so “always” (21.6% versus 12.2%). Although this finding might seem contradictory, a possible explanation is the presence of confounding by exposure or by indication. It is possible that workers with greater patient contact or with high-risk clinical environments report a more frequent practice of hand hygiene due to a greater perception of infection risk. Analytical studies are required to specifically evaluate this hypothesis and allow for a better understanding of the factors associated with MRSA carriage in healthcare personnel.

This study presents four limitations. First, the non-probabilistic convenience sampling constitutes a potential source of selection bias; the personnel who agreed to participate may systematically differ from those who did not, which limits the representativeness of the findings, thus, the reported frequencies describe only the studied group and should not be interpreted as population prevalence. Second, the cross-sectional design captures a single point in time, whereas being a nasal carrier of *S. aureus* can be intermittent or transient. Third, MRSA confirmation was performed only by cefoxitin disk diffusion (EUCAST v14.0, 2024), without molecular tests (PCR for *mecA*) or SCCmec typing; likewise, the observed resistance to linezolid was not confirmed by a reference method, and no D-test was performed for the detection of inducible clindamycin resistance. Future studies should confirm phenotypic linezolid resistance by MIC testing and, when feasible, characterize the genetic determinants (mutations in 23S *rRNA, cfr, optrA, poxtA*). Fourth, practice variables were measured by self-report, which could introduce a potential social desirability bias and overestimate compliance.

In conclusion, in the sample studied, approximately one in ten healthcare workers was a nasal carrier of MRSA. The identification of a higher carrier frequency in certain occupational groups, care areas, and among those who reported less consistent use of PPE highlights the importance of strengthening infection prevention and control measures. These findings provide local evidence on MRSA circulation in healthcare personnel in Ayacucho and can serve as a basis for strengthening institutional strategies for microbiological surveillance, hand hygiene, and appropriate PPE use.
