# Supplementary material for: Frequency of nasal carriers of methicillin-resistant Staphylococcus aureus among health personnel in Ayacucho, Peru
Source: Rev Peru Med Exp Salud Publica. 2026 Jun 16;43(2):197–206. doi: 10.17843/rpmesp.2026.432.15609 (PMC13456926; doi:10.17843/rpmesp.2026.432.15609)
Supplement: Translation

# Translation: Frecuencia de portadores nasales de *Staphylococcus aureus* resistente a la meticilina en personal de salud de Ayacucho, Perú

**Authors:** Alexandra A. Urrutia, Nilda A. Apayco, Sayda L. Cancho, Liz V. Muñoz, Máximo R. Sosa

**Affiliations:** 1 Universidad Nacional de San Cristóbal de Huamanga, Huamanga, Perú

**Keywords:** Portador Sano, *Staphylococcus aureus* resistente a la meticilina, Personal de Salud, Pruebas de Sensibilidad Microbiana, Estudios transversales, Perú

## Abstract

**Objetivo.:**

Estimar la frecuencia de portadores nasales de *Staphylococcus aureus* resistente a la meticilina (SARM) en personal de salud de la Red de Salud Huamanga (Ayacucho, Perú) entre octubre y noviembre de 2024.

**Materiales y métodos.:**

Estudio transversal descriptivo en 265 trabajadores de salud, reclutados mediante muestreo por conveniencia. Los hisopados nasales se cultivaron en agar sal manitol, el SARM se confirmó mediante difusión con disco de cefoxitina (EUCAST v14.0 2024: diámetro <22 mm). La susceptibilidad antimicrobiana se evaluó mediante Kirby-Bauer. El uso de equipo de protección personal (EPP) se registró por cuestionario estructurado. Se calcularon frecuencias absolutas y relativas.

**Resultados.:**

*Staphylococcus aureus* se detectó en 14,0% (37/265) de los participantes. De estos aislamientos, 64,9% (24/37) fueron SARM, lo que representa una frecuencia de 9,1% (24/265). Del total de portadores de *Staphylococcus aureus*, los aislamientos de SARM fueron más frecuentes en internos/practicantes (18,9%; 7/37) y enfermeras (16,2%; 6/37). Los consultorios externos (27,0%; 10/37) y emergencia (13,5%; 5/37) concentraron el mayor número de portadores. Se observó alta resistencia a eritromicina en SARM (70,8%) y en *Staphylococcus aureus* sensible a la meticilina (84,6%).

**Conclusiones.:**

En la muestra estudiada, aproximadamente uno de cada diez trabajadores de salud fue portador nasal de SARM. Dado el muestreo por conveniencia, los hallazgos no deben extrapolarse como prevalencia poblacional. Reforzar el uso de EPP, la higiene de manos y la vigilancia microbiológica son medidas prioritarias para reducir la transmisión.

## INTRODUCCIÓN

La cavidad nasal de individuos sanos, incluido el personal de salud, alberga una diversidad de microorganismos que constituyen la microbiota nasal ^1^. Aunque la mayoría de estos microorganismos son comensales, algunos pueden actuar como patógenos oportunistas bajo determinadas condiciones. Entre estos microorganismos destacan las especies del género *Staphylococcus*, particularmente *S. aureus*, por su capacidad patógena y relevancia clínica ^2^^,^^3^.

*S. aureus* es uno de los principales agentes causales de infecciones tanto en el ámbito nosocomial como comunitario, y resulta especialmente problemático en unidades de cuidados intensivos, servicios neonatales y en pacientes con factores de riesgo como quemaduras, traumatismos, inmunosupresión o neutropenia ^4^^,^^5^. Las infecciones causadas por *S. aureus* pueden originarse por contaminación cruzada a través del personal de salud o por infecciones endógenas derivadas de la propia microbiota del paciente ^6^.

El problema se ha exacerbado significativamente por la aparición de cepas de *S. aureus* resistentes a la meticilina (SARM), lo que complica el tratamiento antimicrobiano e incrementa la morbilidad y mortalidad. En 2017, la Organización Mundial de la Salud clasificó al SARM como uno de los 12 patógenos críticos prioritarios debido a su impacto global en la salud pública ^1^. Los datos epidemiológicos muestran que las infecciones por SARM presentan una tasa de mortalidad 64% mayor en comparación con las causadas por *S. aureus* sensible a la meticilina (SASM) ^7^.

El personal de salud desempeña un papel fundamental en la epidemiología de *S. aureus*, al actuar como reservorio y vector de transmisión, particularmente de cepas multirresistentes ^8^. La colonización nasal asintomática entre trabajadores de salud constituye un factor de riesgo significativo para la diseminación nosocomial, especialmente durante brotes epidémicos ^9^. Estudios previos en América Latina han documentado tasas variables de portadores nasales de SARM en personal de salud, que oscilan entre 5,0% y 53,9% según la región y las características institucionales ^10^^-^^15^.

En el contexto peruano, los datos sobre portadores nasales de SARM en personal de salud se limitan principalmente a dos estudios realizados por Garcia *et al*. en un hospital de tercer nivel de Lima, que reportó una frecuencia de 13,3% entre el personal de los servicios de medicina y cirugía ^12^, y el estudio de Cayllahua Arenas realizado en el Hospital Goyeneche de Arequipa ^16^. No se identificaron estudios previos en zonas andinas como Ayacucho, donde las condiciones socioeconómicas y la disponibilidad de recursos podrían influir en las prácticas de bioseguridad y el control de infecciones, lo que justifica generar evidencia local.

El objetivo del presente estudio fue estimar la frecuencia de portadores nasales de SARM en personal de salud de la Red de Salud Huamanga, Ayacucho y describir el perfil de resistencia antimicrobiana de los aislamientos recuperados.

**MENSAJES CLAVE**

Motivación del estudio. El *Staphylococcus aureus* resistente a la meticilina (SARM) es un patógeno crítico prioritario según la Organización Mundial de la Salud y su transmisión nosocomial implica al personal de salud como reservorio. La información local en Ayacucho, Perú es escasa, dificultando el control basado en evidencias.

Principales hallazgos. En 265 trabajadores, la frecuencia de portadores nasales de SARM fue 9,1% (24/265). Los internos/practicantes (18,9%) y los consultorios externos (27,0%) concentraron los mayores portadores nasales. La resistencia a eritromicina alcanzó 70,8% en SARM y 84,6% en *Staphylococcus aureus* sensible a la meticilina.

Implicancias en salud pública. Este estudio proporciona datos descriptivos locales para la Red de Salud Huamanga que pueden servir como punto de partida para vigilancia institucional.

## MATERIALES Y MÉTODOS

### Diseño del estudio y población

Se realizó un estudio descriptivo entre octubre y noviembre de 2024 en el personal de salud de la Red de Salud Huamanga, Ayacucho, Perú. El estudio incluyó personal de establecimientos públicos de salud de dos niveles de atención: centros de salud de primer nivel (categorías I-3 y I-4), que brindan atención ambulatoria para condiciones de baja a moderada complejidad, y un hospital de segundo nivel (categoría II-E), que brinda atención ambulatoria y hospitalaria.

El tamaño muestral se estimó mediante la fórmula de proporción para población finita, considerando los siguientes parámetros: población total N = 800 trabajadores de la Red; nivel de confianza del 95% (Z = 1,96); proporción esperada p = 0,133, basada en la frecuencia de portadores nasales de SARM reportada en personal de salud por Garcia *et al*. en un hospital terciario de Lima ^12^; q = 1 − p = 0,867; y un margen de error e = 0,05. El cálculo se realizó en una hoja de cálculo de Microsoft Excel® v.2021, arrojando un tamaño mínimo requerido de 146 participantes.

De un total de 800 trabajadores potencialmente elegibles, 313 otorgaron consentimiento informado por escrito; tras aplicar los criterios de exclusión (no aceptaron participar o no estuvieron disponibles durante el periodo de estudio, uso de antibióticos en 6 meses previos, afecciones médicas que afecten la mucosa nasal o embarazo), 265 fueron incluidos en el análisis final (figura 1), superando el tamaño mínimo requerido.

**Figura 1:** Diagrama de flujo de selección de participantes.

### Variables

Las variables incluidas en este estudio se detallan a continuación, agrupadas por categoría.

*Variable principal*

La condición de portador nasal de SARM se definió operacionalmente como la recuperación de una o más colonias con morfología compatible con *S. aureus* en agar sal manitol (colonias amarillas con acidificación del medio), confirmadas mediante pruebas positivas de catalasa y coagulasa, y con un diámetro de zona de inhibición frente a cefoxitina (30 µg) <22 mm según los puntos de corte EUCAST v14.0 2024. La clasificación fue cualitativa (portador/no portador); la recuperación de al menos una colonia confirmada fue suficiente para clasificar a un participante como portador.

*Variables sociodemográficas*

Edad (años cumplidos; recategorizada en ≤33 y ≥34 años), sexo (masculino/femenino). La categorización de la edad se basó en los datos (división por la mediana).

*Variables ocupacionales*

Se recolectó información sobre el rol/profesional de los participantes, clasificándolos como médicos, enfermeras tituladas, técnicos en enfermería, obstetras, biólogos, odontólogos, psicólogos, farmacéuticos, trabajadores sociales, personal administrativo, personal de limpieza e internos/practicantes. Estos últimos correspondieron a estudiantes de los últimos años de carreras de ciencias de la salud, como medicina, enfermería, obstetricia, odontología y biología, que realizaban actividades asistenciales bajo supervisión como parte de su internado clínico. Asimismo, se registró el área de trabajo, categorizada en emergencia, sala de operaciones, consultorio externo, laboratorio, farmacia, hospitalización y otros servicios, que incluyeron control de crecimiento y desarrollo (CRED), triaje e inmunización. Finalmente, el tiempo de experiencia laboral se agrupó en tres categorías: menos de un año, entre uno y cinco años y más de cinco años.

*Variables conductuales y de exposición*

Estas variables se incluyeron porque la literatura las identifica consistentemente como factores asociados a portadores nasales de *S. aureus* en personal de salud (uso de EPP, higiene de manos, contacto con dispositivos médicos invasivos, contacto con áreas de alto riesgo) ^12^^,^^18^. Estas variables se recolectaron mediante un cuestionario estructurado de autoinforme previamente probado en cinco trabajadores de salud no incluidos en el análisis para verificar su comprensibilidad: Frecuencia de uso de equipo de protección personal (EPP) (ordinal de 4 niveles: «Siempre», «Frecuentemente», «A veces», «Rara vez»); frecuencia de higiene de manos (ordinal de 3 niveles: «Siempre», «Frecuentemente», «A veces»); uso de mascarilla, guantes y bata (sí/no); uso de desinfectante de manos a base de alcohol (sí/no); contacto reportado con áreas de alto riesgo de exposición microbiológica (sí/no); y contacto reportado con dispositivos médicos invasivos (sí/no).

### Procedimientos microbiológicos

*Recolección de muestras, cultivo e identificación*

Los hisopados nasales se obtuvieron de ambas fosas nasales utilizando un único hisopo estéril con punta de algodón y mango de madera. El hisopo se introdujo suavemente y se rotó en cada fosa nasal para asegurar un muestreo adecuado ^17^. Las muestras se colocaron en tubos Falcon estériles de 15 mL que contenían 1 mL de solución salina estéril al 0,9% y se transportaron al laboratorio (Laboratorio de Inmunología y Microbiología Clínica [Y-207] de la Universidad Nacional de San Cristóbal de Huamanga) en un contenedor térmico mantenido aproximadamente a 4 °C. El tiempo máximo entre la recolección de la muestra y la siembra no superó las 5 horas. No se utilizó caldo de preenriquecimiento; las muestras se sembraron directamente en agar sal manitol (ASM) mediante el método de estriado en placa. Las placas se incubaron a 37 °C y se evaluaron a las 18 h; aquellas con crecimiento escaso o equívoco se reincubaron y reevaluaron a las 24 h. Los cultivos sin crecimiento característico después de 72 h se consideraron negativos. Los aislamientos se consideraron *S. aureus* si produjeron colonias amarillas con cambio de color del medio (fermentación de manitol), y se confirmaron como positivos mediante pruebas de catalasa y coagulasa ^19^. Un mínimo de una colonia (≥1 UFC) con morfología típica confirmada por pruebas bioquímicas fue suficiente para clasificar a un participante como portador.

*Pruebas de susceptibilidad antimicrobiana*

Los aislamientos de *S. aureus* se evaluaron en agar Mueller-Hinton mediante el método de difusión con disco de Kirby-Bauer. Las suspensiones bacterianas se estandarizaron al patrón de turbidez 0,5 de McFarland y las placas se incubaron a 37 °C durante 18 h. La susceptibilidad antimicrobiana se evaluó utilizando Multodiscs Staph (Liofilchem®, ref. 95260), un sistema de discos multiantibiótico que contiene los siguientes agentes: cefoxitina, levofloxacino, netilmicina, tetraciclina, eritromicina, gentamicina, rifampicina y linezolid. Los diámetros de las zonas de inhibición se midieron e interpretaron de acuerdo con los puntos de corte clínicos de las guías del European Committee on Antimicrobial Susceptibility Testing (EUCAST) v14.0 (2024). El control de calidad se realizó utilizando *S. aureus* ATCC 25923 como cepa de referencia para difusión con disco, siguiendo las recomendaciones EUCAST. Los diámetros de zona para cada antibiótico se verificaron dentro de rangos aceptables antes de interpretar los resultados de los aislamientos clínicos. La resistencia a la meticilina (SARM) se confirmó fenotípicamente según el resultado del disco de cefoxitina; los aislamientos con diámetro de zona de inhibición <22 mm se clasificaron como SARM, de acuerdo con los criterios EUCAST 2024. No se realizó prueba D (eritromicina/clindamicina) para detección de resistencia inducible a clindamicina.

### Análisis estadístico

El procesamiento y análisis de los datos se realizó utilizando IBM SPSS Statistics for Windows, versión 27.0.1 (IBM Corp., Armonk, NY, Estados Unidos). Las variables cuantitativas se resumieron mediante medidas de tendencia central y dispersión, mientras que las variables categóricas se describieron mediante frecuencias absolutas (n) y relativas (%). Considerando el corte transversal del estudio y la estrategia de selección de participantes, los resultados se presentan como una descripción de la muestra incluida, sin realizar análisis inferenciales.

### Consideraciones éticas

El estudio fue aprobado por el Comité Institucional de Ética de la Universidad Nacional de San Cristóbal de Huamanga, mediante Constancia N.° 001-2025/CEI-VRI-UNSCH-Ayacucho. El estudio se realizó en conformidad con los principios éticos de la Declaración de Helsinki para investigación en seres humanos. Se obtuvo consentimiento informado por escrito de todos los participantes. La confidencialidad de los datos se aseguró mediante codificación anonimizada y bases de datos protegidas con contraseña.

## RESULTADOS

De los 800 trabajadores de salud contactados, 313 otorgaron consentimiento informado; después de aplicar los criterios de exclusión, 265 fueron incluidos en el análisis final. La condición de portador nasal de *S. aureus* se observó en el 14,0% (37/265). La edad promedio fue de 36,1 años (rango 19-69), con predominio del sexo femenino (77,0%). El 41,5% tenía menos de un año de experiencia laboral. Los internos/practicantes mostraron la mayor frecuencia de portadores nasales de *S. aureus* con relación al total de participantes (4,2% de la muestra total, 11/265), mientras que no se observaron portadores nasales entre farmacéuticos, trabajadores sociales y personal de limpieza (tabla 1).

**Tabla 1:** Características sociodemográficas, ocupacionales y de bioseguridad de la muestra estudiada, según estado de portadores nasales de S. aureus.

Característica		Total (n=265)	S. aureus + (n=37)	S. aureus - (n=228)
		n	n (%)	n (%)
Sexo				
	Femenino	204	29 (14,2)	175 (85,8)
	Masculino	61	8 (13,1)	53 (86,9)
Edad (años)				
	≤33	135	21 (15,6)	114 (84,4)
	≥34	130	16 (12,3)	114 (87,7)
Rol/profesión				
	Interno/practicante	72	11 (15,3)	61 (84,7)
	Enfermera	46	7 (15,2)	39 (84,8)
	Técnico en enfermería	37	6 (16,2)	31 (83,8)
	Obstetra	22	5 (22,7)	17 (77,3)
	Médico	21	1 (4,8)	20 (95,2)
	Biólogo	19	1 (5,3)	18 (94,7)
	Odontólogo	13	3 (23,1)	10 (76,9)
	Farmacéutico	12	0 (0,0)	12 (100,0)
	Administrativo	9	1 (11,1)	8 (88,9)
	Psicólogo	7	2 (28,6)	5 (71,4)
	Trabajador social	5	0 (0,0)	5 (100,0)
	Personal de limpieza	2	0 (0,0)	2 (100,0)
Área de trabajo				
	Emergencia	51	7 (13,7)	44 (86,3)
	Sala de operaciones	1	0 (0,0)	1 (100,0)
	Consultorio externo	84	13 (15,5)	71 (84,5)
	Laboratorio	37	4 (10,8)	33 (89,2)
	Farmacia	14	0 (0,0)	14 (100,0)
	Hospitalización	7	3 (42,9)	4 (57,1)
	Otrosa	71	10 (14,1)	61 (85,9)
Frecuencia de uso de EPP				
	Siempre	137	17 (12,4)	120 (87,6)
	Frecuentemente	93	10 (10,8)	83 (89,2)
	A veces	28	7 (25,0)	21 (75,0)
	Rara vez	7	3 (42,9)	4 (57,1)
Frecuencia de higiene de manos				
	Siempre	230	28 (12,2)	202 (87,8)
	Frecuentemente	26	8 (30,8)	18 (69,2)
	A veces	9	1 (11,1)	8 (88,9)
Otras prácticas (Sí)				
	Uso de desinfectante de manos	253	34 (13,4)	219 (86,6)
	Uso de mascarilla, guantes y bata	133	13 (9,8)	120 (90,2)
	Contacto con áreas de alto riesgo	17	2 (11,8)	15 (88,2)
	Contacto con dispositivos médicos	90	9 (10,0)	81 (90,0)

EPP: equipo de protección personal.

Los porcentajes están calculados sobre el total de cada fila.

a La categoría Otros incluye control de crecimiento y desarrollo (CRED), triaje e inmunización.

### Distribución según rol/profesión y área de trabajo

Entre los portadores, el 64,9% fue SARM, lo que representa una frecuencia de portadores nasales de 9,1% en total de la muestra estudiada. La tabla 2 caracteriza a los 37 portadores de *S. aureus* según estado SARM/SASM. Los portadores nasales de SARM entre los 37 portadores siguió un patrón similar, con internos/practicantes (7/37, 18,9%) y enfermeras (6/37; 16,2%) como los grupos más representados (tabla 2).

**Tabla 2:** Distribución de portadores de S. aureus según estado S. aureus resistente a la meticilina / S. aureus sensible a la meticilina y características sociodemográficas y ocupacionales (n=37).

Característica		Portadores n	SARM n (%)	SASM n (%)
Sexo				
	Femenino	29	20 (69,0)	9 (31,0)
	Masculino	8	4 (50,0)	4 (50,0)
Edad (años)				
	≤33	21	14 (66,7)	7 (33,3)
	≥34	16	10 (62,5)	6 (37,5)
Rol/profesional				
	Interno/practicante	11	7 (63,6)	4 (36,4)
	Enfermera	7	6 (85,7)	1 (14,3)
	Técnico en enfermería	6	2 (33,3)	4 (66,7)
	Obstetra	5	4 (80,0)	1 (20,0)
	Médico	1	1 (100,0)	0 (0,0)
	Biólogo	1	1 (100,0)	0 (0,0)
	Odontólogo	3	2 (66,7)	1 (33,3)
	Administrativo	1	0 (0,0)	1 (100,0)
	Psicólogo	2	1 (50,0)	1 (50,0)
Área de trabajo				
	Emergencia	7	5 (71,4)	2 (28,6)
	Consultorio externo	13	10 (76,9)	3 (23,1)
	Laboratorio	4	2 (50,0)	2 (50,0)
	Hospitalización	3	3 (100,0)	0 (0,0)
	Otros^a^	10	4 (40,0)	6 (60,0)

SARM: *S. aureus* resistente a la meticilina, SASM: *S. aureus* sensible a la meticilina.

Los porcentajes están calculados sobre el total de cada fila.

a La categoría Otros incluye control de crecimiento y desarrollo (CRED), triaje e inmunización.

### Perfil de resistencia antimicrobiana

Las pruebas de susceptibilidad antimicrobiana revelaron perfiles de resistencia variables entre los aislamientos SARM y SASM (tabla 3). Ambos grupos exhibieron altas tasas de resistencia a eritromicina (70,8% en SARM; 84,6% en SASM). La resistencia a tetraciclina fue mayor en SASM (53,8%) en comparación con SARM (25,0%). En contraste, los aislamientos SASM mostraron mayor resistencia a linezolid (53,8%) en comparación con SARM (29,2%) y gentamicina (29,2% frente a 30,8% en SASM). La resistencia a rifampicina se mantuvo baja en ambos grupos (<10%), mientras que la resistencia a levofloxacino fue similar (29,2% en SARM; 30,8% en SASM).

**Tabla 3:** Comparación de la resistencia antibiótica según antibióticos evaluados entre *S. aureus* resistente a la meticilina y *S. aureus* sensible a la meticilina.

Antibiótico	***S. aureus* resistente a la meticilina (n=24)**		***S. aureus* sensible a la meticilina (n=13)**	
	Resistente n (%)	Sensible n (%)	Resistente n (%)	Sensible n (%)
Linezolid (LNZ)^a^	7 (29,2)	17 (70,8)	7 (53,8)	6 (46,2)
Rifampicina (RD)	2 (8,3)	22 (91,7)	1 (7,7)	12 (92,3)
Gentamicina (CN)	7 (29,2)	17 (70,8)	4 (30,8)	9 (69,2)
Eritromicina (ERY)	17 (70,8)	7 (29,2)	11 (84,6)	2 (15,4)
Tetraciclina (TE)	6 (25,0)	18 (75,0)	7 (53,8)	6 (46,2)
Netilmicina (NET)	3 (12,5)	21 (87,5)	5 (38,5)	8 (61,5)
Levofloxacino (LEV)	7 (29,2)	17 (70,8)	4 (30,8)	9 (69,2)

La susceptibilidad se evaluó mediante difusión con disco de Kirby-Bauer utilizando Multodiscs Staph (Liofilchem®, ref. 95260), interpretada según puntos de corte clínicos EUCAST 2024 (v.14.0).

El SARM se definió por diámetro de zona <22 mm frente a cefoxitina.

Los porcentajes se calculan dentro de cada subgrupo (R + S = 100% para SARM [n=24] y SASM [n=13] por separado).

a Las tasas de resistencia a linezolid no fueron confirmadas por método de referencia (microdilución en caldo / E-test).

### Prácticas de control de infecciones y colonización

Entre los 37 portadores, la distribución de las prácticas de control de infecciones autoinformadas mostró que una mayor frecuencia de portadores reportó uso inconsistente de EPP. Dentro del grupo de internos/practicantes, el 54,5% (6/11) reportó usar siempre EPP (tabla 4).

**Tabla 4:** Frecuencia de uso de equipo de protección personal entre participantes positivos para *S. aureus*.

Rol/profesión	Siempre n (%)	Frecuentemente n (%)	A veces n (%)	Rara vez n (%)	Total n (%)
Interno/practicante	6 (54,5)	2 (18,2)	3 (27,3)	0 (0,0)	11 (29,7)
Técnico en enfermería	4 (66,7)	1 (16,7)	0 (0,0)	1 (16,7)	6 (16,2)
Odontólogo	2 (66,7)	1 (33,3)	0 (0,0)	0 (0,0)	3 (8,1)
Administrativo	0 (0,0)	0 (0,0)	0 (0,0)	1 (100,0)	1 (2,7)
Biólogo	1 (100,0)	0 (0,0)	0 (0,0)	0 (0,0)	1 (2,7)
Enfermera	4 (57,1)	2 (28,6)	1 (14,3)	0 (0,0)	7 (18,9)
Médico	0 (0,0)	0 (0,0)	0 (0,0)	1 (100,0)	1 (2,7)
Obstetra	0 (0,0)	3 (60,0)	2 (40,0)	0 (0,0)	5 (13,5)
Psicólogo	0 (0,0)	1 (50,0)	1 (50,0)	0 (0,0)	2 (5,4)
Total	17 (45,9)	10 (27,0)	7 (18,9)	3 (8,1)	37 (100,0)

Los porcentajes están calculados sobre el total de cada fila y los porcentajes de la columna Total se calculan sobre todos los portadores de *S. aureus* (n=37).

En relación con la higiene de manos, el 75,7% (28/37) de los participantes con aislamiento positivo para *S. aureus* reportó realizarla siempre, el 21,6% (8/37) frecuentemente y el 2,7% (1/37) a veces. Entre las enfermeras, el 85,7% (6/7) indicó realizar siempre la higiene de manos, mientras que fue el 100,0% en biólogos, obstetras y psicólogos (tabla 5).

**Tabla 5:** Frecuencia de higiene de manos entre casos positivos para *S. aureus* según rol/profesión.

Rol/profesión	Siempre n (%)	Frecuentemente n (%)	A veces n (%)	Total n (%)
Interno/practicante	8 (72,7)	3 (27,3)	0 (0,0)	11 (29,7)
Técnico en enfermería	4 (66,7)	2 (33,3)	0 (0,0)	6 (16,2)
Odontólogo	2 (66,7)	1 (33,3)	0 (0,0)	3 (8,1)
Administrativo	0 (0,0)	0 (0,0)	1 (100,0)	1 (2,7)
Biólogo	1 (100,0)	0 (0,0)	0 (0,0)	1 (2,7)
Enfermera	6 (85,7)	1 (14,3)	0 (0,0)	7 (18,9)
Médico	0 (0,0)	1 (100,0)	0 (0,0)	1 (2,7)
Obstetra	5 (100,0)	0 (0,0)	0 (0,0)	5 (13,5)
Psicólogo	2 (100,0)	0 (0,0)	0 (0,0)	2 (5,4)
Total	28 (75,7)	8 (21,6)	1 (2,7)	37 (100,0)

Los porcentajes están calculados sobre el total de cada fila y los porcentajes de la columna Total se calculan sobre todos los portadores de *S. aureus* (n=37).

## DISCUSIÓN

Este estudio describe una frecuencia de portadores nasales de *S. aureus* de 14,0% entre personal de salud de la Red de Salud Huamanga, con una frecuencia de SARM de 9,1% en la muestra estudiada. Asimismo, se observó una mayor frecuencia de portadores nasales de SARM entre internos y practicantes (18,9%), una mayor concentración de portadores en los consultorios externos (27,0%) y entre el personal que reportó un uso inconsistente de EPP.

La frecuencia global de portadores nasales de *S. aureus* (14,0%) fue menor que la reportada en Paraguay (26,6%) ^20^ y Uruguay (24%) ^21^. Las diferencias pueden reflejar características poblacionales, protocolos institucionales de control de infecciones o variaciones metodológicas. Sin embargo, la frecuencia de SARM entre los portadores (64,9%) fue marcadamente mayor que en otros entornos latinoamericanos, lo que sugiere una fuerte presión selectiva antimicrobiana en la región. La frecuencia de portadores nasales de SARM (9,1%) es comparable con Ecuador (12,5%) ^22^, pero menor que Paraguay (23,8%) ^20^. En contraste, estudios en unidades brasileñas de atención primaria reportaron prevalencias superiores al 50% ^10^, posiblemente reflejando diferencias en los niveles de atención o en la carga comunitaria de SARM.

Los internos/practicantes fueron el grupo con la mayor frecuencia de portadores nasales, seguido de enfermeras. Esto concuerda con datos colombianos, donde los internos también fueron el grupo más colonizado (33,8%) ^23^, pero contrasta con estudios argentinos donde predominaron los médicos ^11^. Los factores pueden incluir experiencia limitada en protocolos de bioseguridad, alta carga de contacto directo con pacientes, rotación frecuente entre servicios y supervisión más débil. Las mujeres representaron el 77,0% de los participantes, consistente con la feminización de enfermería y obstetricia en el Perú ^24^^-^^26^. Aunque el 78,4% de los casos positivos fueron mujeres, esto refleja la composición de la fuerza laboral y no una susceptibilidad asociada al sexo.

La adherencia a prácticas básicas de bioseguridad fue subóptima. La Organización Panamericana de la Salud destaca la higiene de manos como la estrategia más eficaz contra el SARM ^27^. En Perú, Cayllahua Arenas ^16^^)^ encontró una menor frecuencia de portadores entre el personal que se lavaba las manos >10 veces por turno. La evidencia también respalda la importancia de la técnica y el uso de clorhexidina y la fricción sin cepillo como determinantes de la reducción de la carga bacteriana ^28^. El bajo cumplimiento entre internos puede relacionarse con la carga laboral, limitaciones de recursos y refuerzo insuficiente, lo que subraya la necesidad de capacitación dirigida dentro de este grupo.

En la muestra estudiada, la frecuencia de SARM se concentró descriptivamente en consultorios externos y servicios de emergencia más que en hospitalización. A diferencia de los estudios que identifican a las unidades de cuidados intensivos como puntos críticos ^29^, esta distribución podría reflejar un alto flujo ambulatorio, la presencia de portadores comunitarios asintomáticos ^30^ o una aplicación diferencial de la bioseguridad entre servicios. Investigaciones venezolanas reportaron mayores portadores en medicina interna y cirugía (30,4%) ^9^, mientras que estudios en Lima mostraron menor prevalencia en pediatría y ginecología (2,1%) en comparación con medicina y cirugía (13,3%) ^12^. La variabilidad entre servicios destaca la necesidad de una vigilancia diferenciada.

Los perfiles de resistencia revelaron una multirresistencia preocupante. Entre los aislamientos SARM, el 70,8% fue resistente a eritromicina, el 29,2% a linezolid, gentamicina y levofloxacino, el 25,0% a tetraciclina, el 12,5% a netilmicina y el 8,3% a rifampicina. Estos hallazgos son consistentes con estudios venezolanos (52,9% de resistencia a eritromicina) ^8^ y brasileños (49,2% de resistencia a eritromicina) ^10^. La alta resistencia a eritromicina tanto en SARM (70,8%) como en SASM (84,6%) sugiere presión selectiva por el uso de macrólidos. Las tasas de resistencia a linezolid en este estudio (29,2% en SARM y 53,8% en SASM) son marcadamente superiores a los datos de vigilancia global que informan resistencia <1% ^31^^,^^32^. Esta discrepancia probablemente refleja factores metodológicos más que resistencia verdadera. CLSI documentó recientemente que los puntos de corte previos de difusión con disco para linezolid en estafilococos producían errores superiores al 15% ^33^. En este estudio, el uso de Multodiscs Staph (Liofilchem® ref. 95260) en lugar de discos colocados individualmente pudo haber comprometido aún más la precisión debido al potencial solapamiento de zonas de inhibición. Estos datos de resistencia a linezolid no deben utilizarse para orientar terapia empírica hasta que sean confirmados mediante microdilución en caldo y caracterización molecular.

Asimismo, se observó una mayor frecuencia de portadores entre el personal que reportó realizar higiene de manos “frecuentemente” en comparación con aquellos que indicaron hacerlo “siempre” (21,6% frente a 12,2%). Aunque este hallazgo podría parecer contradictorio, una posible explicación es la presencia de confusión por exposición o por indicación. Es posible que los trabajadores con mayor contacto con pacientes o con entornos clínicos de alto riesgo reporten una práctica más frecuente de higiene de manos debido a una mayor percepción del riesgo de infección. Se requieren estudios analíticos que evalúen de manera específica esta hipótesis y permitan comprender mejor los factores asociados a portador de SARM en el personal de salud.

Este estudio presenta cuatro limitaciones. Primero, el muestreo no probabilístico por conveniencia constituye una fuente potencial de sesgo de selección; el personal que aceptó participar puede diferir sistemáticamente de quienes no lo hicieron, lo que limita la representatividad de los hallazgos, por lo que las frecuencias reportadas describen únicamente al grupo estudiado y no deben interpretarse como prevalencia poblacional. Segundo, el diseño transversal captura un único punto en el tiempo, mientras que el ser portador nasal de *S. aureus* puede ser intermitente o transitoria. Tercero, la confirmación de SARM se realizó únicamente mediante difusión con disco de cefoxitina (EUCAST v14.0, 2024), sin pruebas moleculares (PCR para *mecA*) ni tipificación SCCmec; asimismo, la resistencia observada a linezolid no fue confirmada mediante método de referencia y no se realizó prueba D para detección de resistencia inducible a clindamicina. Estudios futuros deberían confirmar la resistencia fenotípica a linezolid mediante pruebas de CIM y, cuando sea factible, caracterizar los determinantes genéticos (mutaciones en 23S *rRNA*, *cfr*, *optrA*, *poxtA*). Cuarto, las variables de práctica se midieron mediante autoinforme, lo que podría introducir un potencial sesgo de deseabilidad social y sobrestimar el cumplimiento.

En conclusión, en la muestra estudiada, aproximadamente uno de cada diez trabajadores de salud fue portador nasal de SARM. La identificación de una mayor frecuencia de portador en determinados grupos ocupacionales, áreas de atención y entre quienes reportaron un uso menos consistente de EPP resalta la importancia de fortalecer las medidas de prevención y control de infecciones. Estos hallazgos aportan evidencia local sobre la circulación de SARM en personal de salud de Ayacucho y pueden servir como base para el fortalecimiento de las estrategias institucionales de vigilancia microbiológica, higiene de manos y uso adecuado de EPP.
